# Relaxation and Strain-Hardening Relationships in Highly Rejuvenated Metallic Glasses

**DOI:** 10.3390/ma15051702

**Published:** 2022-02-24

**Authors:** Xudong Yuan, Daniel Şopu, Kaikai Song, Jürgen Eckert

**Affiliations:** 1 Erich Schmid Institute of Materials Science, Austrian Academy of Sciences, Jahnstraße 12, A-8700 Leoben, Austria; xudong.yuan@oeaw.ac.at (X.Y.); juergen.eckert@unileoben.ac.at (J.E.); 2 Fachgebiet Materialmodellierung, Institut für Materialwissenschaft, Technische Universität Darmstadt, Otto-Berndt-Straße 3, D-64287 Darmstadt, Germany; 3 School of Mechanical, Electrical and Information Engineering, Shandong University (Weihai), Weihai 264209, China; songkaikai@sdu.edu.cn; 4 Department of Materials Science, Chair of Materials Physics, Montanuniversität Leoben, Jahnstraße 12, A-8700 Leoben, Austria

**Keywords:** metallic glass, molecular dynamics simulations, rejuvenation, relaxation, strain-hardening

## Abstract

One way to rejuvenate metallic glasses is to increase their free volume. Here, by randomly removing atoms from the glass matrix, free volume is homogeneously generated in metallic glasses, and glassy states with different degrees of rejuvenation are designed and further mechanically tested. We find that the free volume in the rejuvenated glasses can be annihilated under tensile or compressive deformation that consequently leads to structural relaxation and strain-hardening. Additionally, the deformation mechanism of highly rejuvenated metallic glasses during the uniaxial loading–unloading tensile tests is investigated, in order to provide a systematic understanding of the relaxation and strain-hardening relationship. The observed strain-hardening in the highly rejuvenated metallic glasses corresponds to stress-driven structural and residual stress relaxation during cycling deformation. Nevertheless, the rejuvenated metallic glasses relax to a more stable state but could not recover their initial as-cast state.

## 1. Introduction

Metallic glasses (MGs) are obtained by fast cooling from the melt to avoid crystallization and exhibit a disordered structure with higher-energy states [1,2,3]. As-cast MGs are thermodynamically metastable and can spontaneously convert to a lower energy state via aging (relaxation) [4,5]. However, many strategies can push MGs to undergo an opposite process and reach a more disordered state which is called rejuvenation. Rejuvenation can be induced by reheating [6,7,8] and faster quenching [9], thermal cycling [10], elastostatic and heavy plastic deformation [11,12,13,14,15], irradiation [16], etc. Rejuvenation is an effective way to inspire the structure of MGs to restore flexibility with the increase of free volume and enthalpy [17,18,19,20] and it is regarded as a promising approach for tuning the deformability of MGs. It is now seen as a common way to improve the plasticity of MGs [11,21] since it can ameliorate the highly localized deformation mechanism and could ultimately eliminate the formation of critical shear bands [7,11]. Moreover, structural rejuvenation can also provide strain-hardening under certain loading conditions [21].

Although rejuvenation has captured increasing attention due to its scientific significance, the precise control of the degree of rejuvenation in MGs and the design of highly rejuvenated MGs is still a challenge in experimental work. Rejuvenation is usually associated with free volume accumulation that results to structural softening and hardness reduction. Strain softening is the Achilles’ heel of MGs. While strain-hardening is familiar in polycrystalline metals, it is not found in most MGs [22,23]. However, in some particular cases, strain-hardening has been also observed in monolithic MGs. Here, the suppression of shearing through size or geometric constraints, as demonstrated for nanosized samples [24,25] or notched rods [26], may also lead to the apparent strain-hardening. Besides this, extreme rejuvenated MGs could even show strain-hardening that was associated with structural relaxation when loaded in uniaxial tension or compression which can be regarded as a return from the rejuvenated state [21]. Nevertheless, strain-hardening is evaluated with respect to the highly rejuvenated state and the structure never recovers the hardness of the initial as-cast state. Thus, a systematic understanding of the relationship between the degree of rejuvenation and deformation behavior in MGs is missing and an atomistic and mechanistic explanation for experimental observations is required. Additionally, the correlation between structural rejuvenation and the transition from strain-softening to strain-hardening behavior needs to be further studied. Compared to the limitation of the experiment, molecular dynamics (MD) simulations provide useful insights into the rejuvenation and relaxation process of MGs [4,27]. Moreover, MD simulations allows to quantitatively control the fraction of free volume into the glass matrix and, hence, provide a systematic strategy to manipulate the degree of rejuvenation in MGs [28].

In this work, we present MD computer simulations of the deformation behavior of MGs with a controlled degree of rejuvenation. Free volume is homogeneously introduced into the glass matrix by creating vacancies. Uniaxial tensile and compression tests are conducted and the deformation behavior of the rejuvenated glass systems was investigated in comparison to the as-cast MG. In addition, loading–unloading cycling tensile tests are simulated and the mechanism of strain-hardening in the highly rejuvenated MG is highlighted.

## 2. Simulation Details

### 2.1. Samples Preparation

For studying the mechanical properties of the rejuvenated MGs, classical MD simulations were performed using the program package LAMMPS [29] and the python scripting interface. A Cu_64_Zr_36_ glassy system of 48,000 atoms with the dimensions of around 8 × 8 × 12 nm^3^ was used as a prototype material. The system was relaxed at 2000 K for 2 ns to get the chemical homogeneity liquid and then quenched down to 50 K with a constant cooling rate of 10^10^ K/s. The constant pressure and temperature (NPT) ensemble were employed and periodic boundary conditions (PBCs) were applied in all three directions during the quenching process. The interatomic interactions were described by the modified Finnis–Sinclair type potential for CuZr binary alloys proposed by Mendelev et al. [30]. For all simulations a constant integration time step of 2 fs was used. After cooling down to 50 K, the system was equilibrated for 1 ns to get the stable initial as-cast MG structure. We homogeneously introduced free volume and systematically rejuvenate the as-cast MG system by applying a dilution procedure through vacancy creation presented in detail in our previous study [28]. In short, atoms are randomly removed from the initial as-cast structure and the degree of rejuvenation is represented by the percentage of atoms that are removed from the system. During the dilution process, the free volume evolution was analyzed to describe the structural state of the system.

### 2.2. Deformation Tests

In this work, uniaxial tensile and compressive tests were conducted to MGs with different degrees of rejuvenation. The temperature was controlled at 50 K using the NPT ensemble. PBCs are used in all three directions. Before loading, the samples are relaxed for 100 ps and all tests were performed under a constant engineering strain rate of 4 × 10^7^/s. The loads were applied along *z*-direction while the stress in the other two directions is kept as zero. Additionally, recent experimental work has shown that compression-induced rejuvenation goes along with strain-hardening in mechanical deformation. In order to provide further analysis of strain-hardening in rejuvenated MG, loading-unloading cycling tensile tests were applied to the as-cast and highly rejuvenated glass samples that are designed by diluting the as-cast MG. At each cycle, the samples were loaded just before the yielding point and then were spontaneously unloaded to zero stress and further relaxed for 1 ns to get an equilibrium state. The evolution of the free volume and the atomic shear strain for each sample are visualized using the OVITO analysis and visualization software [31].

## 3. Results and Discussions

**Figure 1:**
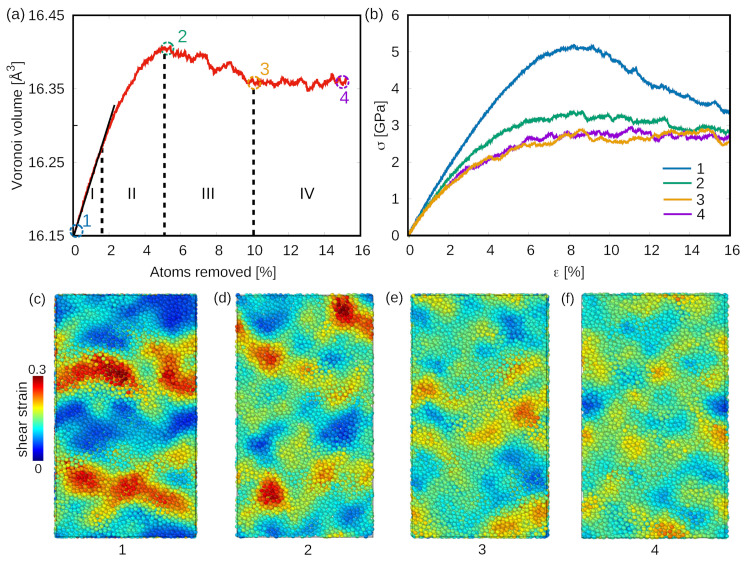
(**a**) The evolution of Voronoi volume during the dilution process. Four samples are picked up at different rejuvenation levels during the dilution process and are marked as sample 1, 2, 3 and 4. (**b**) Stress–strain curves for the four samples during tensile test. (**c**–**f**) The atomic strain map at the strain level of 16% for the four tested samples.

By randomly removing atoms from the as-cast glassy matrix, free volume content increases, which could be correlated to the rejuvenation of MGs [15]. The generation and annihilation of free volume during the dilution procedure were monitored and represented by the Voronoi volume variation which is calculated by the Voronoi tessellation method [19,32,33]. As shown in Figure 1a, four stages of Voronoi volume evolution can be found. In our previous work, the atomic-scale dynamic behavior of the system during the dilution process was investigated [28]. The Voronoi volume linearly increases during stage *I* and slowly increase at stage *II*, illustrating that the free volume continues to be generated inside the glassy matrix during the dilution process [34]. Continuing to remove atoms from the system, the activation of β relaxation and the transit to α relaxation corresponds to a decrease of Voronoi volume as observed in stage *III*. As enough atoms are removed from the glassy system, the dominant α relaxation behavior is activated and the competition between free volume creation and annihilation defines the dynamic balance in the Voronoi volume (Figure 1a stage *IV*). At this stage, the free volume can not be generated in the glassy matrix with further dilution and the rejuvenation reaches a threshold, which can be called the highly rejuvenated state.

It has been previously shown that structure rejuvenation in MGs is an effective strategy to tailor its mechanical properties [7,15,21,35,36,37]. In order to investigate the mechanical properties of the MG systems with different degrees of rejuvenation, the deformation behavior of MGs at different stages of dilution was tested under uniaxial tensile loading. Four samples which are picked from the glassy structure at a different dilution level are chosen as the loading objects (Figure 1a) and the results of the tensile tests are analyzed. Figure 1b shows the stress–strain curves of the four loaded samples where a clear strain softening effect can be seen from sample 1 to sample 4. The higher the rejuvenation level, the lower yield stress and the better plasticity the sample has. For sample 1, a sudden stress drop appears in the plastic deformation region of the stress–strain curve which indicates a strain-softening behavior of the as-cast glass and also reflects the tendency of a highly localized deformation (Figure 1c). The smooth slope in the stress–strain curve of sample 2 compared to sample 1 highlights a more homogeneous deformation mode. At this point, the β relaxation is pronounced due to the excess free volume which can be associated with the activation of a large number of shear transformation zones (STZs) [38,39,40]. Due to the existence of earlier activated STZs, lower stress is needed for the formation of plastic zones, which finally leads to lower yield stress compared to sample 1. Nevertheless, the β relaxation is the locally string-like dynamic behavior of atoms [38,41,42] and, hence, localized deformation can still be seen in sample 2, as highlighted in Figure 1d. Finally, in sample 3 and sample 4, although these two tested systems are picked at different dilution levels, in both, the activated α relaxation is driven by atoms of high mobility. Eventually, a homogeneous deformation mechanism instead of localized shear deformation can be seen in these two samples (see Figure 1e,f).

**Figure 2:**
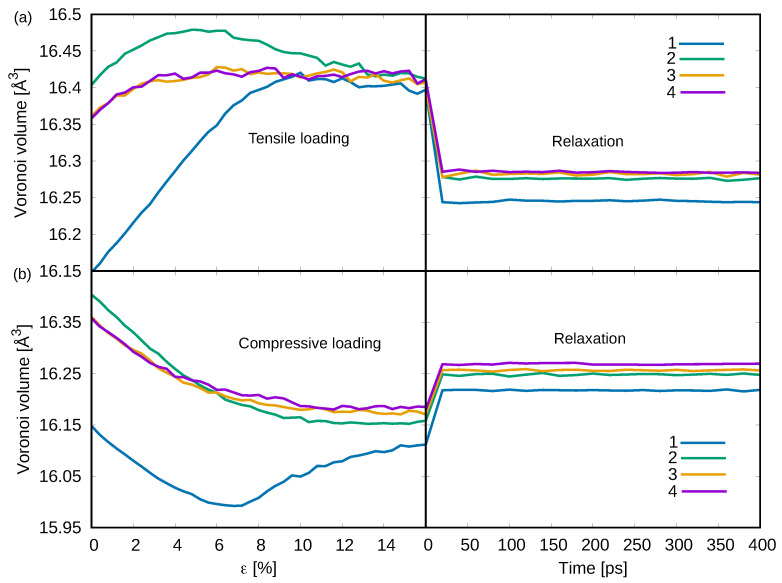
The Voronoi volume evolution for the four tested samples during (**a**) tensile and (**b**) compressive loading and during the unloading and relaxation processes.

The evolution of Voronoi volume with respect to tensile deformation for the four chosen samples is monitored and shown in Figure 2a. Not surprisingly, for sample 1, the Voronoi volume increases significantly with the applied external strain and remains higher even after removing the load indicating a deformation induced rejuvenation process [14,43,44,45,46]. On the contrary, under tensile loading the other three samples with different degrees of rejuvenation (sample 2, sample 3 and sample 4) show initially a slight increase of the Voronoi volume corresponding to elastic stretching of the bonds. However, once the load is released, the Voronoi volume decreases resembling a relaxation process. A similar effect is observed when deforming in compression (see Figure 2b). The Voronoi volume in sample 1 decreases at the early stage of loading due to the system’s elastic shrinkage and then increases indicating a deformation-induced free volume generation process (rejuvenation). It is worth noticing that the Voronoi volume of the rejuvenated samples always decreases during the compression process and, for sample 2, it can further decrease compared to samples 3 and 4 when the strain level overcomes 6%. As we mentioned above, samples 3 and 4 are highly rejuvenated MGs, and thus have lower yield stress compared to sample 2, and they can show homogeneous viscous flow without the formation of any localized shear bands during plastic deformation. Contrary to samples 3 and 4, sample 2 is in a more ordered state with a higher fraction of short-range order clusters (SRO) and also has higher contents of free volume (Figure 1a). This explains, on one hand, the higher Voronoi volume value before the compression test. On the other hand, a higher fraction of SRO means a higher yield stress that allows the system to compress (elastically deform) to a lower volume before yielding. The evaluation of Voronoi volume during deformation and unloading indicates that the as-cast MGs always rejuvenate under deformation while, on the contrary, the extreme rejuvenated systems exhibit constantly structural relaxation. Nevertheless, one should mention that there is still a marginal difference between the values of Voronoi volume after conducting tensile and compression cycling loading. After deformation, the glassy systems contain both elastic strains (expand/tensile strains and shrinkage/compressive strains) and plastic strains. When the applied stress is released, the elastic strains are mostly released but there are always some residual stresses confined into the glassy matrix [47].

**Figure 3:**
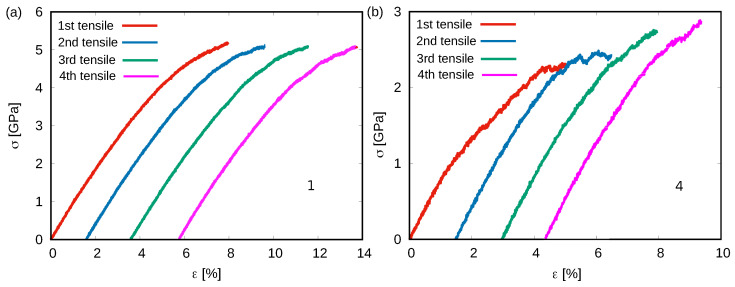
The stress–strain curves of the loading–unloading tensile test of (**a**) the as-cast MG (sample 1) and (**b**) the highly rejuvenated MG (sample 4).

**Figure 4:**
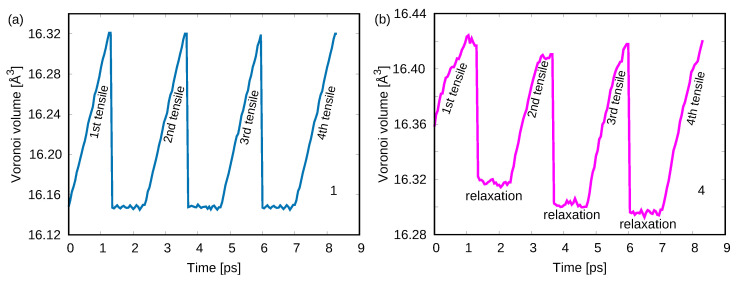
Voronoi volume evolution during cycling tensile test of (**a**) the as-cast MG (sample 1) and (**b**) the highly rejuvenated MG (sample 4).

Previous studies have shown that extreme rejuvenated MGs could even exhibit strain-hardening when loaded in uniaxial tension or compression [21]. Hence, we correlate the return from the rejuvenation states during loading to the experimentally observed strain-hardening. To do this, the highly rejuvenated MG (sample 4) is uniaxially deformed before yielding, and is then unloaded-reloaded several times. The same deformation process was applied to the as-cast MG (sample 1) for comparison. Figure 3 shows the stress–strain curves of the cycling tensile tests for the two samples. As expected, the stress–strain curves in Figure 3a indicate that the yield stress of sample 1 keeps a constant value at each reloading. Contrary to sample 1, Figure 3b shows that the yield stress of sample 4 gets higher on successive loadings, which indicates strain-hardening. As we discussed above, the rejuvenated MG exhibits relaxation during cycling deformation, which could be the reason for the observed strain-hardening. In order to prove this, the Voronoi volume evolution during the whole process for the two samples is monitored and shown in Figure 4. During each time of loading, the Voronoi volume of sample 1 linearly increases due to the elastic expansion caused by the applied stress. After releasing the applied stress, the volume can immediately decrease to its unloaded value. No extra free volume generation or annihilation after each cycle is observed indicating no structural fluctuations. As for sample 4, the Voronoi volume also increases with the applied stress during reloading. However, it is worth noting that at each time of unloading stage, the Voronoi volume can decrease to a lower value compared to its last unloaded state, which reveals a stress-driven relaxation behavior. Under given deformation conditions, an MG can approach a steady-state energy [7]. The highly rejuvenated MG has a higher initial energy state and contains a higher fraction of free volume (see Figure 1a) compared to the as-cast MG. The excess free volume could be released via a stress-driven relaxation turning to a more stable state that, in the end, causes the observed strain-hardening behavior in highly rejuvenated MG.

**Figure 5:**
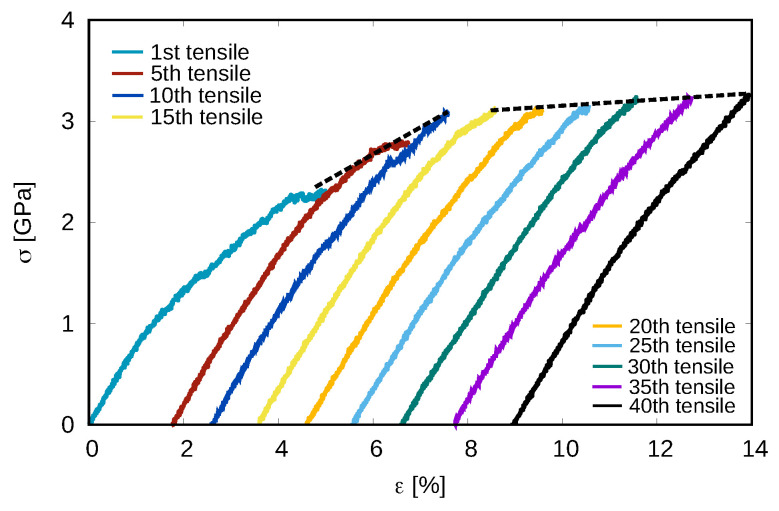
The evolution of stress–strain curves of the highly rejuvenated MG from the 1st to 40th reloading tensile tests. The dashed lines display the two regimes of the strain-hardening behavior.

**Figure 6:**
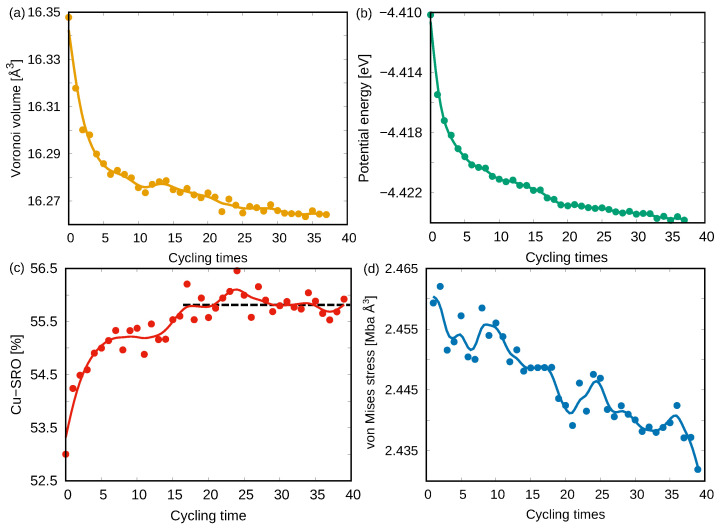
Evolution of (**a**) the Voronoi volume, (**b**) the potential energy, (**c**) the fraction of Cu–SRO, and (**d**) the von Mises stress of the highly rejuvenated MG during the 40 times cycling tensile tests. All of the parameters were calculated at each unloading stage.

As the cycling loading proceeds, the stress–strain curves show strain-hardening behavior up to the 40th reloading test (see Figure 5). This is supported by the continuous decrease of the free volume and the potential energy observed for sample 4 (see Figure 6a,b). The correlation between the variations in the atomic-level structure of the glassy system and the observed strain-hardening can be quantified using the fraction of the most favored Cu-centered short-range order (Cu-SRO) clusters (Figure 6c). As expected, the fast increases of the Cu-SRO fraction from 1st to 15th cycles demonstrate a continuous structural relaxation process that reverts the system towards a more ordered state. Interestingly, the linear-like variation of Cu-SRO fraction from the 15th to 40th cycles indicates that there is no further structural relaxation. This suggests that the stress-driven structural relaxation is not the only reason for the observed strain-hardening in the rejuvenated MGs during the cycling tensile. Since residual stresses also can be responsible for the efficient strain-hardening in MGs [47], the internal stress state of the rejuvenated MG during each unloading stage was monitored and represented by the von Mises stress (VMS). As shown in Figure 6d, the decrease of VMS during the whole cycling process indicates that the residual stress, which was generated during the initial dilution process, is slowly released.

The highly rejuvenated MG, sample 4, is in a high-energy state that possesses a high fraction of disordered structures and, at the same time, contains residual stresses. At the early stage of cycling loading (before the 15th cycle), stress-induced structural relaxation pushes the system towards a more ordered state and leads to significant strain-hardening behavior (Figure 5). After, the structural relaxation becomes sluggish (Figure 6c) but residual stresses still exist in the glass. Therefore, as the cycling loading continues (after 15th cycle), the residual stresses are still released (Figure 6d) during each loading stage that slightly lowers the excess free volume and the potential energy of the system (Figure 6a,b) and, consequently, increases the yield stress at the next cycle. Although the stress relaxation during the unloading–reloading process continues even after the 40th cycle, it is worth noticing that the hardening effect would level off and the rejuvenated MG could not achieve the strength of the as-cast state. Hence, one may reasonably predict that cycling loading can never relax a highly rejuvenated MG back to its initial as-cast state (at least after a reasonable number of cycles). To overcome shear-softening and achieve “real” strain-hardening and extensive ductility, one may shift focus to stress modulated MGs with residual stresses [47].

## 4. Conclusions

To summarize, in the present work, by randomly removing atoms from monolithic MGs, glass structures with different degrees of rejuvenation were designed and mechanically tested. Contrary to the observed rejuvenation behavior in the as-cast glassy system, the extreme rejuvenated MG exhibited structural and stress relaxation during uniaxial deformation. Under cycling tensile loading, the highly rejuvenated MG showed strain-hardening mainly for two reasons: (1) The effect of stress-induced structural relaxation that progressively decreases the potential energy and free volume. (2) As the structural relaxation becomes sluggish, the unloading–reloading process continues to release internal residual stresses from the glass that, over progressive cycling loading, induce further strain-hardening. The incremental relaxation process during the cycling loading–unloading tests leads the rejuvenated MG towards a more stable state. Nevertheless, no evidence shows that it can recover the hardness of the initial as-cast state even after 40 cycling tensile tests.

## Data Availability

All data regarding the simulation and modeling are available on request.
